# A Retrospective Evaluation of Spleen Hydatid Cyst Treatments: The Spleen-Preserving Conservative Approach is Preferable to Splenectomy

**DOI:** 10.5152/eurasianjmed.2022.20367

**Published:** 2022-06-01

**Authors:** Ercan Korkut, Nurhak Aksungur, Gürkan Öztürk

**Affiliations:** 1 Department of General Surgery, Atatürk University Faculty of Medicine, Erzurum, Turkey

**Keywords:** Echinococcosis, spleen, cysts, splenectomy, cystotomy

## Abstract

**Objective:** Cystic echinococcosis (CE) is rarely encountered in the spleen, even in areas where the disease is endemic. There is no consensus in the literature concerning the treatment of splenic cystic echinococcosis. This study was intended to compare the treatment results and factors affecting the selection of the spleen-preserving approach or splenectomy in treatment.

**Materials and Methods:** Four hundred and seventy patients operated for cystic echinococcosis at a university clinic between January 2010 and December 2019 were retrospectively evaluated. Splenic cystic echinococcosis was identified in 22 patients (4.7%). Demographic features as well as clinical and laboratory findings of patients who underwent splenectomy or spleen-preserving operations were compared between the groups.

**Results:** Spleen-preserving cystotomy was performed on 18 patients (81.8%), and splenectomy on 4 (18.2%). Eleven patients (50%) had isolated cystic echinococcosis in the spleen, while another 11 (50%) had hepatic and splenic cystic echinococcosis. The median cyst size was 10.0 cm (min: 3, max: 20). Splenectomy patients had significantly larger cyst sizes (median, min-max; 8.0 cm, 3-15 cm vs. 15.0 cm, 10-20) (Z = 2.071, *P* = .042). Postoperatively, 1 patient from the splenectomy group (25.0%) developed deep vein thrombosis, and another from the cystotomy group (5.5%) developed pulmonary embolism. No other serious complications, re-operation requirements, or recurrence were observed after a median follow-up period of 27.0 months (min: 5, max: 92).

**Conclusion:** Since no collections or recurrence were observed, and splenic functions were preserved in patients who underwent spleen-preserving cystotomy, we recommend the use of spleen-protective cystotomy. Splenectomy should be employed in patients with cyst diameters exceeding 10 cm, with a central location, and in cases with difficult cavity management and a high risk of developing postoperative collections.

**Main Point**

- Cystic echinococcosis (CE) disease is rarely seen in the spleen.
- Therefore, there is a limited number of publications in the literature on the treatment of splenic CE and there is no consensus on the treatment of splenic CE in the literature.
- In many publications, splenectomy is recommended for the curative treatment of splenic CE. However, we do not scientifically accept this approach for all splenic CE patients.
- In our study, we compare the spleen-preserving and splenectomy cases and explain that spleen-preserving treatment should be performed in surgical treatment.

## Introduction

The most common cause of echinococcosis is *Echinococcus granulosus*, a small cestode. This parasite lives in the canine intestines. Eggs shed in feces cause disease in the larval form in the intermediate host via contaminated foods. Although it can be observed in any organ, hydatid disease is most commonly seen in the liver (60%-70%), lung (15%-20%), and spleen (0.9%-8%).^1^

There is no specific finding on the onset of splenic cystic echinococcosis (CE). Cysts usually grow slowly, 0.3-2 cm annually.^1^ As the cyst grows, compression of the surrounding tissues can cause upper left quadrant pain. Diagnosis is usually made incidentally during imaging performed for other reasons. Serological methods can be used for confirmation. However, symptoms may change when the liver and lungs are involved. Neglected large cysts can be perforated, in which case, the patient may experience acute abdominal symptoms and anaphylactic reactions.

Splenic CE often presents together with hepatic CE. However, it can also rarely be seen as an isolated disease in the spleen.^2-4^ Due to its low risk and low recurrence rate, splenectomy is recommended in some publications as a primary surgical method in splenic CE.^3-7^ However, there is no consensus regarding the treatment of splenic hydatid cysts.^1,8^ Current developments in interventional radiology have permitted this condition to be treated percutaneously. However, most articles on this subject are based on case reports and limited clinical trials.

The purpose of this article is to evaluate the results of splenectomy and spleen-preserving approaches in our clinic in the treatment of hydatid cyst disease of the spleen.

## Materials and Methods

Four hundred and seventy patients operated for diagnoses of hydatid cyst at the Atatürk University Medical Faculty General Surgery Clinic, Turkey, between January 2010 and December 2019 were evaluated retrospectively. Twenty-two patients were diagnosed with splenic CE. Patients were assessed in terms of age, sex, ultrasonography, computed tomography (CT) serology, presence of cysts in other organs, the surgical method applied, duration of surgery, postoperative complications, length of hospital stay, and cyst recurrence rates.

The diagnosis was based on radiological imaging methods, such as CT and ultrasound (US). In cases in which no diagnosis could be established, serological tests, including the indirect hemagglutination test, and the enzyme-linked immunosorbent assay were applied. All cysts were classified according to the World Health Organization (WHO) classification.

All patients were started on albendazole tablets at 10 mg/kg 15 days before surgery, continuing for 2 months after surgery. Patients were invited for regular monthly check-ups and were closely monitored with physical examination and laboratory values against the toxic effects of albendazole.

All patients were operated by the same surgical team. The choice of incision was based on the presence of cysts in the liver.

The cyst circumference was protected with compresses impregnated with 0.04% chlorhexidine gluconate (Chx-Glu) in order to prevent contamination of the cyst contents into the surrounding tissues. The cyst content was aspirated before the procedure, and its pressure was reduced. Next, 0.04% Chx-Glu solution was injected into the cyst. After waiting for 5 minutes, the cyst was opened and aspirated.

Abdominal US imaging was performed before discharge. Patients were followed-up using US at the 3rd and 12th months after surgery. However, abdominal CT was employed in cases with diffuse cysts.

The study was approved by the Ethics Committee of Atatürk University (07.05.2020, B.30.2.ATA.0.01.00/207). Informed consent was obtained from all individual participants included in the study.

### Statistical analysis

The data were analyzed on Statistical Package for the Social Sciences (SPSS) version 25 software (IBM SPSS Corp.; Armonk, NY, USA). The results were presented as frequencies, percentages, median, and range. The Mann–Whitney *U* test was used to compare numerical data between 2 groups, and Fisher’s exact test was applied for categorical variables. A *P*-value of <.05 was considered statistically significant.

## Results

Splenic cysts were present in 22 patients in our series, 10 (45.5%) males and 12 (55.5%) females. The median age of the patients was 41.5 (25-65) years. Eleven (50%) patients had hepatic and splenic hydatid cysts, while the other 11 (50%) had isolated splenic cysts. Single cysts were observed in 20 patients (90.9%) and double cysts in 2 (9.1%).

The majority (63.6%, n = 14) of the cysts were type CE1 according to the WHO classification. No type CE4 or type CE5 cysts were observed. The median cyst size was 10.0 (3-20) cm.

Nineteen (86.4%) patients underwent open surgery, while 3 (13.6%) underwent laparoscopic surgery. Splenectomy was performed on 4 patients (18.1%). No intraoperative or postoperative blood transfusion was required in any case. Patients scheduled for splenectomy were vaccinated against pneumococcal and *Hemophilus influenza* infections before surgery. The median length of hospital stay was 7.5 (5-23) days, with a median postoperative follow-up length of 27.0 (5-92) months. No recurrence was observed in any patient during the follow-up period. Patients’ descriptive findings are summarized in Table 1.

The spleen-preserving approach was more frequently employed for class CE1 patients. In addition, postoperative complications were more common in splenectomy patients (Table 2). No mortality occurred in any patient.

Splenectomized patients were younger, had larger cyst sizes, and were followed up for longer than individuals who underwent spleen-preserving operations. Operative times and lengths of hospital stay were not significantly different between these 2 groups (Table 3).

## Discussion

The spleen is the third most common site of hydatid disease after the liver and lung. Splenic involvement has been reported in between 0.9% and 8% of cases of hydatid cyst disease.^1,9^ In our series, 22 out of 470 patients who underwent surgery had splenic cysts (4.6%). A search of our hospital’s radiology clinic records revealed that only 1 out of 490 patients treated with the percutaneous method in the same time period underwent percutaneous treatment for the splenic cyst. The rate of hydatid cyst in the spleen was 2.2% among all 960 patients diagnosed with CE and who underwent surgical and percutaneous procedures in our hospital.

Since the liver and lung are the organs most commonly affected by hydatid disease (90%), splenic hydatid cysts usually accompany the condition in these organs. Eleven of the 22 patients in our series had CE in the liver, while 11 had isolated splenic CE. There were no cases with combined hydatid cysts of the lung and spleen.

As with hepatic CE disease, the latent period in splenic CE is long. The disease does not lead to any immediate specific findings. The symptoms it produces depend on the size of the cyst and the involvement of other organs. Patients with isolated splenic hydatid cysts most often report left upper quadrant pain. Neglected large cysts can perforate spontaneously or as a result of trauma. These cases may present with acute abdomen. Additionally, an anaphylactic reaction may develop as a result of perforation. However, no splenic cyst perforation was observed in any patient in our series.

Diagnosis of hydatid disease is usually based on US and CT. However, simple cysts, pseudocysts, splenic abscesses, epidermoid cysts, and cystic neoplasms causing cystic lesions in the spleen can produce similar manifestations of splenic hydatid cysts. Differential diagnosis of these patients should be established using magnetic resonance imaging and immunological-serological tests.^1,9^ Imaging methods were mostly sufficient for diagnosis in this study, and serological tests were not requested from any patient.

Hydatid disease generally involves 1 hydatid cyst located in the spleen, although more than 1 lesion in the spleen has been described in the literature. Two patients in our series had 2 cysts, while the other patients had single cysts.

Medical therapy can be attempted in isolated small spleen cysts. None of the patients in the present study received medication alone. Prophylactic albendazole (10 mg/kg) was administered to all patients with surgical treatment.

No recurrence was observed in any patient in the postoperative period. During surgery, 0.04% chlorhexidine gluconate was used as a scolicidal agent to inactivate the cyst contents and prevent scattering to surrounding tissues. In an experimental controlled rat study, Puryan et al^10^ concluded that 0.04% chlorhexidine gluconate is a highly effective and non-toxic agent. We also think that 0.04% chlorhexidine gluconate is a highly potent scolicidal agent in terms of inactivating the contents of the cyst and preventing contamination of the surrounding tissue. No chemical peritonitis was observed in any patient using 0.04% chlorhexidine gluconate.

Different techniques are applied in hydatid disease of the liver as well as the spleen. Percutaneous treatment, splenic conservative surgical methods (cystotomy unroofing omentoplasty, partial splenectomy, or enucleation), and total splenectomy have been recommended by some authors.^2,7,8^ However, studies also state that the most appropriate method in hydatid cyst of the spleen should be selected on a case-by-case basis depending on the location of the cyst, its size, WHO-Gharbi typing, and the presence of cysts in other organs.^8,11-13^

Percutaneous treatment can be employed in splenic hydatid cyst disease, especially for class CE1 and CE3A cysts less than 5 cm in size.^12,14^ Percutaneous treatment is not usually applied to CE2, CE3B, CE4, and CE5 patients. The rate of cavity shrinkage is less in CE2 and CE3B patients with multivesicular cysts. The risk of recurrence and abscess formation is higher with the percutaneous treatment in these patients.^14,15^ In a study conducted by Akhan et al.^14^ PAIR (puncture, aspiration, injection, respiration) was administered to 8 out of 12 patients, while catheters were applied to 4 individuals with class CE1 and CE3A hydatid cysts. No complications were observed in 7 patients, abscess developed in 4, and splenectomy was performed on 2 due to abscess. Although 490 patients with hepatic CE underwent percutaneous treatment in our interventional radiology clinic, percutaneous intervention was performed on 1 patient with a type CE1 cyst in the spleen. Percutaneous treatment is not favored in splenic hydatid cyst in our hospital due to risks such as spreading into the abdomen, bleeding, and abscess compared to hepatic hydatid cysts.

Controlling hemostasis is challenging due to the risk of excessive bleeding of the spleen tissue in partial splenectomy. Large cysts may be attached to the surrounding tissues. It is difficult to perform partial splenectomy and enucleation without bursting the cyst in this patient group. Partial splenectomy was not performed in any cases in our clinic.

Susceptibility to infection and post-splenectomy sepsis may be seen following splenectomy, especially in children. Appropriate prophylactic measures should be taken in cases scheduled for splenectomy, and vaccination against pneumococci and *H. influenza* should be applied 15 days before surgery. None of the patients who underwent splenectomy in our clinic experienced postoperative sepsis or severe infection.

Spleen-protective conservative cystotomy was performed in 18 out of 22 patients in our clinic. Omentoplasty was not performed in any case, although it is recommended in some publications in order to reduce bile fistula and shrink the residual cavity in hepatic hydatid cysts.^16,17^ Vagainus et al^18^ reported no difference in recurrence between cases with or without omentoplasty. Omentoplasty was not performed in the present study since there was no risk of bile fistula in the splenic hydatid cysts, and residual cavity management was performed with energy devices. The size of the cyst, its proximity to the splenic hilus and vessels, adhesion to the surrounding tissue, and the type of cyst based on the WHO classification are crucial for the selection of the surgical treatment method in splenic hydatid cysts,. Arikanoglu et al^8^ reported that 8 out of 11 patients with splenic CE underwent splenectomy, 2 underwent cystotomy, and enucleation was performed on 1. The mean cyst diameter in that study was 8.6 cm. The authors stated that the cysts were located peripherally in 8 patients, centrally in 2 patients, and in the hilum in 1. That study also reported no difference between patients who underwent spleen-preserving intervention and those who underwent splenectomy.

Spleen-preserving surgery was predominantly performed in our clinic. However, it is difficult to dissect large cysts (generally >10 cm) in the hilum from the splenic hilar vascular structures, thus making successful un-roofing and cavity drainage impossible. Although not our favorite approach, patients with large hilar cysts in whom safe dissection was not possible underwent splenectomy. The operative time is somewhat longer in these patients since the cyst is large and adheres to the surrounding tissue. In patients who underwent spleen-preserving surgery, unroofing was performed with energy devices for cavity management after evacuating the cyst. No collection was observed in any patient after surgery. Patients who underwent spleen-sparing surgery had a shorter operative time, less risk of bleeding, and a simpler procedure than splenectomy. No recurrence was observed in any patient in the postoperative follow-up.

In spleen hydatid cyst disease, spleen-preserving methods and splenectomy can be performed laparoscopically. Three patients in our clinic with isolated type CE1 and type CE3A cysts underwent laparoscopic surgery. Patients who undergo laparoscopic surgery will have shorter operating times and shorter hospital stays than those undergoing open surgical procedures.

Bleeding may occur in patients who have undergone splenectomy. These patients are at increased lifetime risk of bacterial infection, venous thromboembolism (VTE), and pulmonary embolism (PE) compared to other patients. Thrombocytosis is common in splenectomy patients. Additionally, postoperative VTE and PE are reported in patients undergoing splenectomy.^19-21^ Thomsen et al^22^ reported that the risk of VTE in patients undergoing splenectomy increased 19.8-fold compared to the general population, while the risk of PE increased 32.6-fold. Although VTE developed in 1 of the 4 patients who underwent splenectomy in our series, PE occurred in 1 of the 18 patients who underwent spleen-sparing surgery. The spleen-preserving approach reduces the lifetime risk of VTE and infection.

In conclusion, some publications recommend splenectomy as the most appropriate surgical option in the treatment of splenic hydatid cyst. However, in our clinical experience, no postoperative collection in the short-term and no recurrence in the long-term were observed in spleen-preserving conservative cystotomy cases. We think cystotomy is a simpler and safer method than splenectomy and is the one which preserves splenic function. Operative time in cystotomy is also shorter in cases with isolated splenic cysts. No thrombocytosis occurs after splenectomy in patients undergoing cystotomy and the risk of thromboembolism and deep vein thrombosis is lower. We suggest that splenectomy should be employed in patients with a cyst diameter exceeding 15 cm, with a central location, and in cases with difficult cavity management, a high risk of developing postoperative collections, and with less splenic tissue. Percutaneous procedures are less advised in the spleen than in hepatic hydatidosis due to the risk of intra-abdominal spread, bleeding, and abscess. We mostly adopt a conservative clinical approach to hydatid cysts, and we recommend spleen-preserving surgery.

## Figures and Tables

**Table 1.** Descriptive Findings of the Participants

		n	%	Median	Min.	Max.
Number of cysts in the spleen	1	20	90.9			
2	2	9.1			
Localization	Peripheral	17	77.3			
Hilar	5	22.7			
WHO class type	CE1	14	63.6			
	CE2	4	18.2			
	CE3A	2	9.1			
CE3B	2	9.1			
Cysts in other organs	Spleen only	11	50.0			
Spleen + liver	11	50.0			
Surgical procedure	Cystotomy + unroofing	15	68.2			
	Laparoscopic cystotomy	3	13.6			
Splenectomy	4	18.2			
Complications	Fever	3	13.6			
	Dyspnea	4	18.2			
	Pulmonary embolism	1	4.5			
	None	13	59.1			
DVT	1	4.5			
Cyst diameter (cm)				10.0	3.0	20.0
Duration of the operation (min)				92.5	50.0	140.0
Duration of hospitalization (days)				7.5	5.0	23.0
Duration of follow-up (days)				27.0	5.0	92.0

CE, cystic echinococcosis; DVT, deep vein thrombosis.

**Table 2.** Comparison of Categorical Variables Between Patients Undergoing Splenectomy and Spleen-Preserving Interventions

		Surgical Procedure					
		Cystotomy + unroofing		Splenectomy	
n	%	n	%	Chi^2^	*P* ^*^
Sex	Male	8	44.8	2	50.0	0.041	1.000
Female	10	55.6	2	50.0		
Number of cysts in the spleen	1	16	88.9	4	100.0	0.489	1.000
2	2	11.1	0	0.0		
Localization	Peripheral	17	94.4	0	0.0	16.622	.001
Hilar	1	5.6	4	100.0		
WHO class type	CE1	14	77.8	0	0.0	11.519	**.006**
	CE2	3	16.7	1	25.0		
	CE3A	1	5.6	1	25.0		
CE3B	0	0.0	2	50.0		
Cysts in other organs	None	8	44.4	3	75.0	1.222	.586
Liver	10	55.6	1	25.0		
Complications	Fever	1	5.6	2	50.0	11.131	**.010**
	Dyspnea	3	16.7	1	25.0		
	Pulmonary embolism	1	5.6	0	0.0		
	DVT	0	0.0	1	25.0		
None	13	72.2	0	0.0		

^*^Fisher’s exact test.

CE, cystic echinococcosis; DVT, deep vein thrombosis.

P: <.005 statistically significant.

**Table 3.** Comparison of Numerical Variables Between Patients Undergoing Splenectomy and Spleen-Preserving Interventions

	Surgical procedure						Z^*^	*P*
	Cystotomy + unroofing			Splenectomy				
Median	Min.	Max.	Median	Min.	Max.
Age	46.0	28.0	65.0	32.0	25.0	40.0	2.003	**.042**
Cyst diameter (cm)	8.0	3.0	15.0	15.0	10.0	20.0	2.071	**.042**
Duration of the operation (min)	90.0	50.0	140.0	107.5	100.0	125.0	1.718	.087
Duration of hospitalization (days)	7.5	5.0	23.0	7.5	6.0	13.0	0.131	.902
Duration of follow-up (days)	20.5	5.0	82.0	81.5	49.0	92.0	2.643	**.005**

^*^ Mann–Whitney *U* test

P: <.005 statistically significant.
